# Synthetic high-density lipoprotein (sHDL): a bioinspired nanotherapeutics for managing periapical bone inflammation

**DOI:** 10.1038/s41368-024-00316-w

**Published:** 2024-07-02

**Authors:** Renan Dal-Fabbro, Minzhi Yu, Ling Mei, Hajime Sasaki, Anna Schwendeman, Marco C. Bottino

**Affiliations:** 1 Department of Cariology, Restorative Sciences, and Endodontics, School of Dentistry, University of Michigan, Ann Arbor, MI USA; 2 Department of Pharmaceutical Sciences, College of Pharmacy and Biointerfaces Institute, University of Michigan, Ann Arbor, MI USA; 3 Department of Biomedical Engineering, College of Engineering, University of Michigan, Ann Arbor, MI USA

**Keywords:** Pulpitis, Root canal treatment

## Abstract

Apical periodontitis (AP) is a dental-driven condition caused by pathogens and their toxins infecting the inner portion of the tooth (*i.e*., dental pulp tissue), resulting in inflammation and apical bone resorption affecting 50% of the worldwide population, with more than 15 million root canals performed annually in the United States. Current treatment involves cleaning and decontaminating the infected tissue with chemo-mechanical approaches and materials introduced years ago, such as calcium hydroxide, zinc oxide–eugenol, or even formalin products. Here, we present, for the first time, a nanotherapeutics based on using synthetic high-density lipoprotein (sHDL) as an innovative and safe strategy to manage dental bone inflammation. sHDL application in concentrations ranging from 25 µg to 100 µg/mL decreases nuclear factor Kappa B (NF-κB) activation promoted by an inflammatory stimulus (lipopolysaccharide, LPS). Moreover, sHDL at 500 µg/mL concentration markedly decreases in vitro osteoclastogenesis (*P* < 0.001), and inhibits IL-1α (*P* = 0.027), TNF-α (*P* = 0.004), and IL-6 (*P* < 0.001) production in an inflammatory state. Notably, sHDL strongly dampens the Toll-Like Receptor signaling pathway facing LPS stimulation, mainly by downregulating at least 3-fold the pro-inflammatory genes, such as *Il1b*, *Il1a*, *Il6*, *Ptgs2*, and *Tnf*. In vivo, the lipoprotein nanoparticle applied after NaOCl reduced bone resorption volume to (1.3 ± 0.05) mm^3^ and attenuated the inflammatory reaction after treatment to (1 090 ± 184) cells compared to non-treated animals that had (2.9 ± 0.6) mm^3^ (*P* = 0.012 3) and (2 443 ± 931) cells (*P* = 0.004), thus highlighting its promising clinical potential as an alternative therapeutic for managing dental bone inflammation.

## Introduction

Inflammation, a fundamental immune response component, is crucial in various human diseases.^1^ While inflammation is a natural defense mechanism against harmful stimuli such as pathogens, toxins, or injury, dysregulated or chronic inflammation can damage tissue and contribute to the pathogenesis of numerous diseases.^1,2^ Conditions ranging from cardiovascular diseases, autoimmune disorders, and metabolic syndromes to neurodegenerative diseases and cancer are associated with inflammation.^3,4^ Besides that, oral inflammation represents a localized manifestation that, if left untreated, can lead to severe complications, including tooth loss and systemic health consequences.^5^

Apical periodontitis (AP) is a condition characterized by inflammation of periradicular tissues caused in response to persistent microbial infection within the root canal system of the affected tooth.^6^ This dental pulp contamination by bacteria and their byproducts evoke nonspecific inflammatory responses, as well as specific immunological reactions in the periradicular tissues, leading to the periapical tissue’s destruction by various chemical mediators that independently or cooperatively modulate the proteolytic activity and the activation of the bone resorption mechanisms.^7–11^ Therefore, AP originates from the host’s immune defense, characterized by infiltrates of various inflammatory cells (T lymphocytes, B lymphocytes, and macrophages) against the action of microorganisms and their products.^7,12^ While there have been significant advancements in endodontic devices and techniques for clinical treatment, there is limited research on substances that can affect the development or healing of AP through the host’s reaction.

High-density lipoprotein (HDL) is an endogenously produced, ultrasmall (8–12 nm in diameter), biodegradable, and safe nanocarrier naturally produced in vivo with a high maximum tolerated dose (up to 8 g of protein when infused).^13^ HDL is known as the “good” cholesterol as it helps to remove excess cholesterol from the bloodstream and transport it to the liver for processing and removal. However, the endogenous HDL has an even broader spectrum of activity, acting by scavenging and neutralizing the pathogen-associated molecular patterns (PAMPs) lipoteichoic acid (LTA) and the lipopolysaccharide (LPS) toxin, inhibiting the Toll-like receptor 4 (TLR4) dependent inflammatory response to LPS derived from multiple bacterial species, as presented in the endodontic infections.^14^ Moreover, some studies also suggested that by modulating the microenvironment of lipid rafts, HDL may inhibit the trafficking of TLR4 to cellular membranes and decrease the activation of downstream inflammation pathways.^15^ The inhibitory effects of HDL on TLR4-dependent inflammatory response suggested the therapeutic potential of HDL on AP. When the overexpressed TLR4 in AP disorder recognizes the LPS released by necrotic endodontic microorganisms, a downstream inflammatory pathway is initiated, leading to the activation of NF-κB and inducing the transcription of cytokines such as TNF-α and interleukin-1β (IL-1β), important ones for triggering a series of inflammatory reactions and promoting bone resorption during the AP development.^16^

With the recent development of synthetic HDL (sHDL), presenting a similar size and mimicking endogenous HDL’s structure and functionality, an enormous field for clinical application has been unlocked, with many research studies and clinical trials using it against both sepsis and atherosclerosis.^17–20^ These reports showed that sHDL could bind to LPS and neutralize its pro-inflammatory effects; in addition to enhancing the clearance of LPS from the bloodstream by promoting its uptake and degradation by immune cells such as macrophages and neutrophils.^21–24^ Due to a lack of high-innovative and safe drugs to improve endodontic treatment, we described for the first time the use of sHDL, an endogenously-mimicking molecule, for the treatment of apical periodontitis.

## Results

### Preparation and characterization of sHDL

**Fig. 1:**
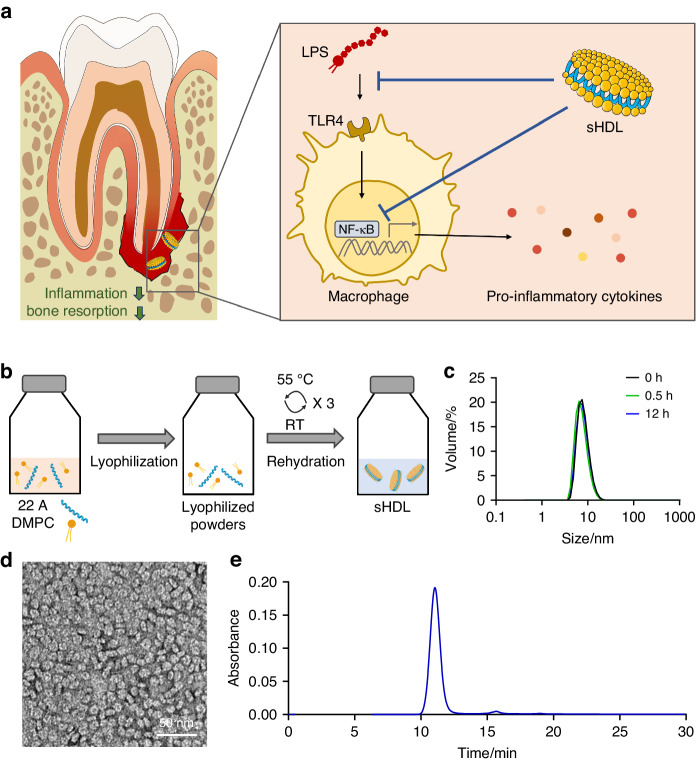
Mechanism of action, preparation, and characterization of sHDLs. **a** Schematic overview depicting the mechanism of action of sHDL in reducing inflammation and bone resorption. **b** Schematic illustration of the preparation process of sHDL. DLS (**c**) and TEM (**d**) showed a particle size of around 10 nm. **e** GPC chromatogram indicated a uniform size distribution of sHDL particles

sHDL nanoparticles were prepared with an ApoA-1 mimetic peptide, 22A, and DMPC using a colyophilization-rehydration method (Fig. 1b). Dynamic light scattering (DLS) analysis showed a particle size around 10 nm, which is similar to the particle size of endogenous HDLs (Fig. 1c). When incubated at physiological temperature, sHDL nanoparticles showed little change in particle size following short term and long term incubation (Fig. 1c), suggesting favorable formulation stability. The particle size of sHDL was further confirmed with transmission electron microscopy (TEM) imaging (Fig. 1d). Gel permeation chromatography (GPC) results suggested a uniform size distribution of sHDL particles (Fig. 1e).

### sHDL inhibits NF-κB activation by LPS

**Fig. 2:**
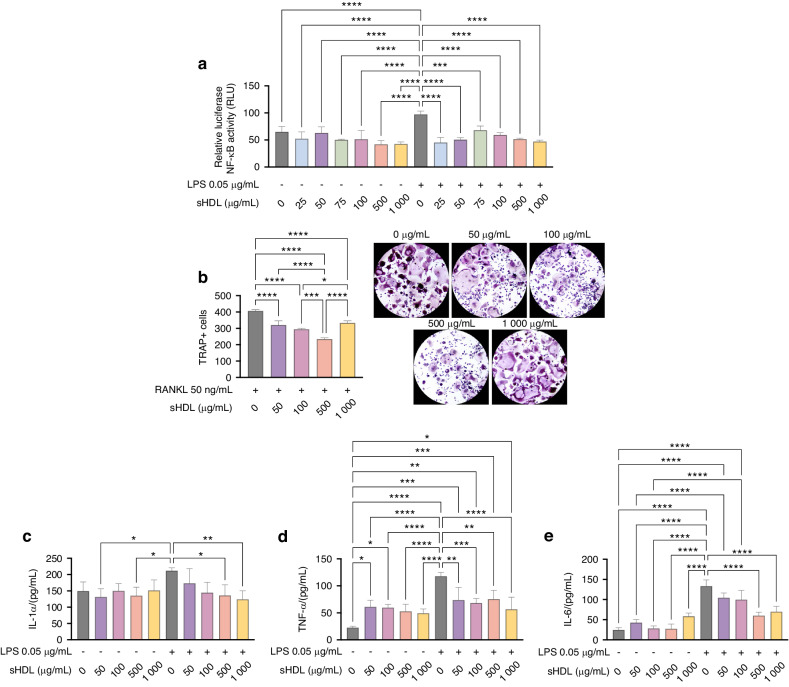
sHDL exposure displayed an immunomodulatory effect compared with RAW264.7 cells stimulated with LPS. **a** NF-κB Luciferase reporter assay showing that sHDL inhibited LPS-stimulated NF-kB activation regardless of the concentration used. **b** Also, the nanoparticle showed the optimal concentration of 500 µg/mL to inhibit osteoclastogenesis in vitro. Moreover, (**c**–**e**) sHDL inhibited the production of IL-1α, TNF-α, and IL-6, showing the suppressed downstream signaling related to the non-activation of NF-kB. Data presented as mean ± standard deviation, one-way ANOVA, and Tukey’s post hoc test, *P ≤ 0.05; **P ≤ 0.01; ***P ≤ 0.001; ****P ≤ 0.000 1. The experiment was conducted with a sample size of *n* = 6 and carried out independently twice

The NF-κB pathway regulates gene expression in response to pathogen stimuli that impact inflammation, immunity, and cell proliferation. To assess the effect of the proposed sHDL nanoparticle on NF-κB, we utilized an NF-κB reporter assay to measure the pathway activity.^25–27^ As expected, after 6 h of LPS stimulation, it significantly enhanced NF-κB transcriptional activity, as evidenced by increased luciferase activity in an NF-κB reporter assay. Notably, increased concentrations of sHDL in an LPS-free environment did not evoke significant NF-κB activation. However, when applied together, the sHDL, in concentrations ranging from 25 µg to 1000 µg/mL, inhibited the NF-κB activation produced by LPS, regardless of the lipoprotein concentration (Fig. 2a).

### sHDL decrease in vitro osteoclastogenesis

RANKL-induced osteoclastogenesis analysis is a powerful tool for studying the factors that regulate osteoclast differentiation and activity and can be used to identify potential targets for drug therapy in bone diseases, such as apical periodontitis. Using sHDL in the RANKL-induced assay significantly decreased osteoclast formation dose-dependently for the 50 µg, 100 µg, and 500 µg/mL compared to the control (RANKL only). When we raised the sHDL concentration to 1 000 µg/mL, the dose-dependently decreased pattern ceased, and a spike in osteoclast formation was noticed, significantly higher than 500 µg and 100 µg/mL concentrations (Fig. 2b). However, although there was an increase in osteoclastic formation compared to lower doses, this number was still significantly lower than the control group, attesting to how sHDL plays an important role in maintaining bone homeostasis by inhibiting osteoclastogenesis.

### Higher concentrations of sHDL inhibit IL-1α, TNF-α, and IL-6 production in an LPS-induced state

Together, IL-1α, TNF-α, and IL-6 are known as the “major pro-inflammatory cytokines” and are often used as biomarkers for inflammation in clinical settings. Using the Enzyme-Linked Immunosorbent Assay, we investigated the effect of sHDL administration in increasing concentrations, 50 µg–1 000 µg/mL, in modulating the proinflammatory effect evoked by LPS treatment on RAW 264.7 cells, as well as the inherent sHDL behavior in the same cell lineage. After 24 h of co-culture, the higher concentrations of sHDL (500 µg and 1000 µg/mL) significantly decreased the production of all three pro-inflammatory cytokines when stimulated with LPS (Fig. 2c–e). For the TNF-α (Fig. 2d), sHDL alone at lower concentrations (50 µg and 100 µg/mL) increased the protein level compared to untreated cells. However, similar TNF-α levels were found by the same sHDL doses when the LPS was applied compared to non-LPS treatment, testifying to its anti-inflammatory potential.

### sHDL modulates the Toll-Like Receptor signaling pathway facing LPS stimulation

**Fig. 3:**
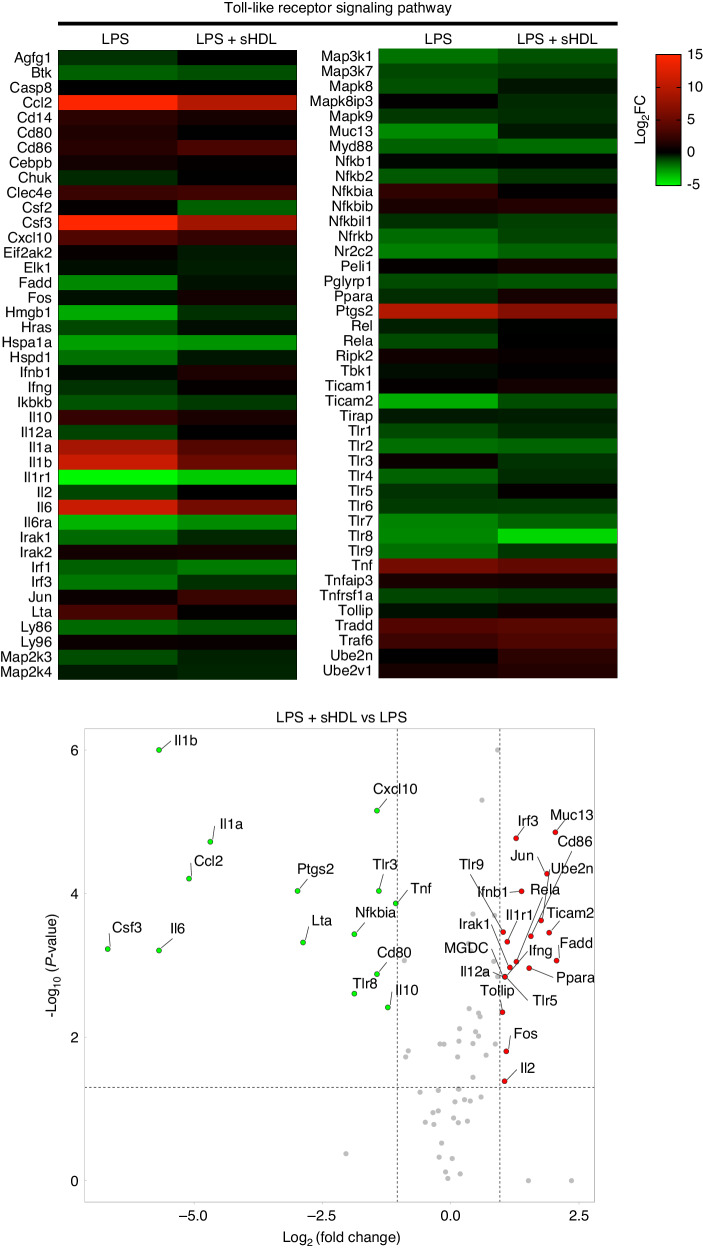
Heatmap generated from 84 genes expression on RAW 264.7 cells quantified using a PCR array for Toll-Like Receptor Signaling Pathway showing the fold change (Log_2_) in the LPS and LPS + sHDL groups, normalized to control (untreated RAW 264.7 cells). Each colored band represents the expression of a single gene from a sample, with higher expression in red and lower expression in green. Volcano plot depicting the Log_2_ fold changes in gene expression between LPS + sHDL vs LPS against t-test *p*-values. Thresholds for fold-change (vertical lines, 2-fold) and significant difference (horizontal line, *P* < 0.05). The more significant the gene expression difference, the more extreme its point will lie on the Log_2_ (fold change) axis. The more significant the difference, the smaller the p-value and, thus, the higher the -Log_10_ (*P*-value). The experiment was conducted with a sample size of *n* = 3 and carried out independently twice

Through the RT^2^ Profiler PCR Array, we evaluated the TLR pathway, crucial for innate immunity, using a system combining real-time PCR and microarray gene expression detection. TLR activation prompts the production of pro-inflammatory cytokines, aiding infection clearance and tissue repair. Assessing this pathway is vital for understanding host-pathogen interactions and developing targeted therapies for infections and inflammatory diseases. The expression profile of the 84 genes relevant to TLR, normalized to control (untreated RAW 264.7 cells), was graphed on a heatmap (Fig. 3). From eighty-four target genes evaluated, the administration of sHDL led to a significant downregulation of fourteen genes (*Il1b, Il1a, Ccl2, Csf3, Il6, Ptgs2, Lta, Cxcl10, Tlr3, Tnf, Nfkbia, Cd80, Tlr8*, and *Il10*), and a significant upregulation of nineteen targets (*Muc13, Irf3, Cd86, Jun, Ube2n, Rela, Il12a, Ticam2, Fadd, Ppara, Ifnb1, Il1r1, Ifng, Tlr9, Irak1, Tlr5, Tollip, Fos, Il2*) compared to non-sHDL LPS-treated cells (Fig. 3).

### sHDL after NaOCl irrigation reduced bone resorption equivalently to the currently standardized treatment

**Fig. 4:**
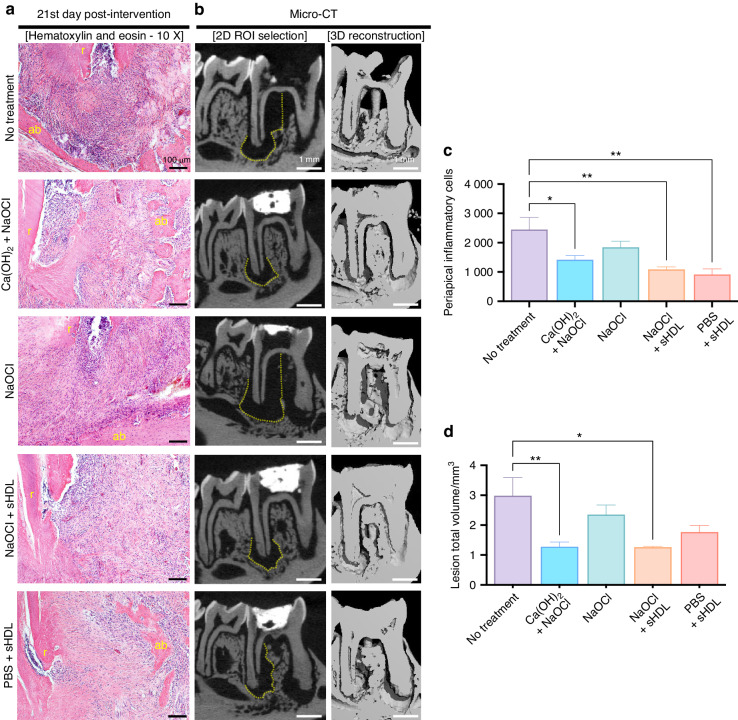
In vivo assessment of inflammation and bone resorption. **a** Histologic findings around tooth apex and (**b**) micro-computed tomography images from the representative sample in each group. **c**, **d** Total periapical inflammatory cell count (H&E) and lesion volume (in mm³, micro-CT). Both sHDL treatment groups (combined with NaOCl or PBS) showed a reduced inflammatory profile around the tooth apex 21 days post-intervention compared to the non-treated group. Additionally, using sHDL after NaOCl irrigation resulted in a bone resorption pattern similar to the benchmark treatment (Ca(OH)_2_ + NaOCl), with significantly smaller lesions localized around the tooth apex than no treatment. Data are presented as mean ± standard deviation. Statistical analysis was performed using one-way ANOVA followed by Tukey’s post hoc test. Significance levels are indicated as *P ≤ 0.05 and **P ≤ 0.01. The sample size was n = 6 per group. In the figure, “r” denotes the root, “ab” denotes the alveolar bone, and the yellow dashed line represents the region of interest (ROI) for lesion total volume quantification on micro-CT

Micro-CT evaluation can provide a detailed analysis of the extent of the lesion, including the size, shape, and location. In the present study, the standardized procedure for root canal treatment in more than one appointment when apical periodontitis is present had the best results. This combination of sodium hypochlorite irrigation plus calcium hydroxide dressing decreased the bone resorption to values of (1.3 ± 0.4) mm^3^, like that displayed by sodium hypochlorite irrigation plus sHDL as dressing (1.3 ± 0.05) mm^3^ (Fig. 4c, d). Although there was no difference between these two treatments, our novel sHDL nanoparticle performed as effectively as the benchmark material, statistically better than SHAM (non-treated tooth), which displayed (2.9 ± 0.6) mm^3^ (*P* = 0.012 3). Notably, this improvement was not due to sodium hypochlorite irrigation since the group using only it was not statistically lower than SHAM.

### sHDL lessened the inflammatory reaction after apical periodontitis treatment

Counting inflammatory cells in histologic slides is crucial for analyzing tissue inflammation, especially in apical periodontitis, where effective treatment typically reduces inflammatory cell counts. In our investigation model, the sHDL nanoparticle groups had the lowest values of inflammatory cell counting, specifically 1090 ± 184 cells when NaOCl was used before and (916 ± 423) cells when PBS was applied previously, both statistically lower than the SHAM group (2 443 ± 931) cells, *P* = 0.004 and *P* = 0.001, respectively. Moreover, they did not differ from the Ca(OH)_2_ + NaOCl group, which had (1 419 ± 378) cells and better results than SHAM. Interestingly, NaOCl alone (1 846 ± 184) could not promote a decrease in the inflammatory profile compared to the noon-treated tooth, once again exhibiting the anti-inflammatory potential of sHDL when applied after NaOCl.

**Fig. 5:**
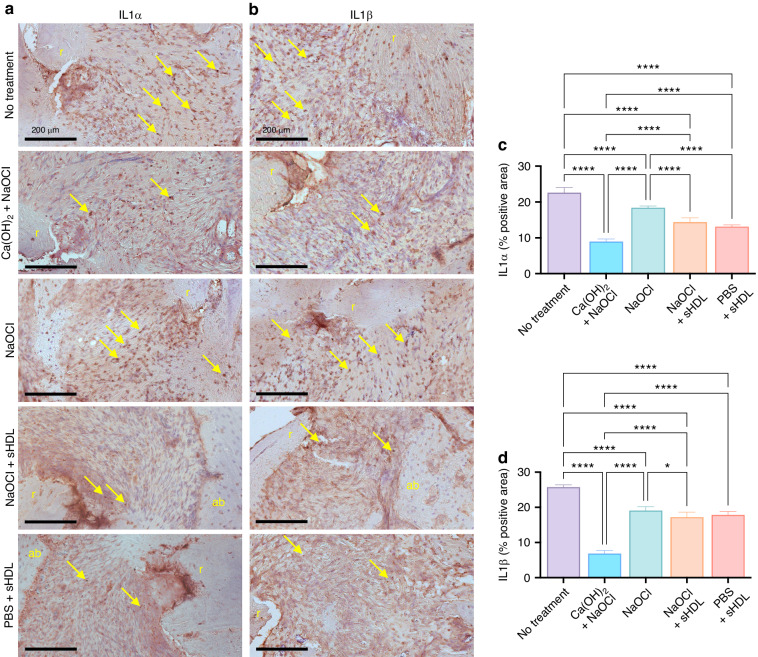
In vivo immunolabeling of pro-inflammatory cytokines. **a**, **b** Representative immunohistochemistry images for IL-1α and IL-1β. **c**, **d** Percentage of positive area DAB-stained for the assigned antibodies. Compared to treatment using only NaOCl irrigation, the combination of NaOCl and sHDL significantly improved anti-inflammatory effects by substantially reducing the expression of IL-1α and IL-1β 21 days post-intervention. However, regardless of the irrigation substance used before sHDL application, the nanoparticle did not achieve the lower levels seen with Ca(OH)_2_ + NaOCl treatment. Data are presented as mean ± standard deviation. Statistical analysis was performed using one-way ANOVA followed by Tukey’s post hoc test, with significance levels indicated as *P ≤ 0.05 and ****P ≤ 0.000 1. The sample size was *n* = 6 per group. In the figure, “r” denotes the root, “ab” denotes the alveolar bone, and the yellow arrow points to immunoreactive cells. Hematoxylin counterstaining was used, and the original magnification was 20×

IL-1α and IL-1β, released by macrophages, lymphocytes, and dendritic cells, are two pro-inflammatory cytokines that play a significant role in developing and progressing apical periodontitis by activating osteoclasts and promoting bone resorption. Also, IL-1α and IL-1β stimulate the production of other pro-inflammatory cytokines, such as TNF-α and IL-6, which amplify the inflammatory response. In our study, by immunolabeling IL-1α and IL-1β, we demonstrated that sHDL nanoparticles used, either after PBS or NaOCl irrigation, significantly decreased their expression compared to SHAM. Moreover, compared to a treatment using only NaOCl irrigation, NaOCl + sHDL improved the anti-inflammatory effects by substantially reducing the expression of IL-1α and IL-1β. However, regardless of the irrigation substance used before the sHDL application, the nanoparticle could not reach the lower values that Ca(OH)_2_ + NaOCl achieved (Fig. 5).

## Discussion

Root canal treatment is a reliable and effective dental procedure for apical periodontitis, a common inflammatory disease of the tooth pulp and surrounding tissues.^28^ Despite new technologies and techniques, such as advancements in endodontic files and intracanal medications, which have improved success rates, the fundamental concept of root canal treatment for apical periodontitis has remained essentially unchanged over the past few decades.^29,30^ This procedure aims to eliminate the source of infection by removing the diseased tissue and filling the root canal with an inert material to prevent further bacterial growth.^31^ Therefore, there are pathways to new materials to be developed, especially those like endogenous human compounds, as they can facilitate healing and tissue regeneration while reducing the risk of infection.

Developing drugs that resemble endogenous molecules is crucial due to their higher biocompatibility with the human body since these compounds are more likely to be recognized and utilized by the body in a beneficial way that minimizes adverse effects. Moreover, these drugs can facilitate the study of biological pathways and mechanisms, ultimately leading to a better understanding of the underlying biology of a disease and more targeted therapies. Thus, developing endogenous-like drugs is a promising strategy that can lead to safer, more effective, and more targeted therapies.^13^ In this study, we developed and investigated the effect of an HDL-mimicking compound that imitates the structure and function of endogenous natural HDL, using it for apical periodontitis treatment. The murine RAW 264.7 cell line was chosen for in vitro assays evaluating endodontic inflammation over primary cell lines due to its consistent and reproducible nature, stable genetic background, and ease of culture. These factors ensure standardized experimental conditions and reliable results while enabling researchers to perform more experiments with less effort. These cells respond to pro-inflammatory stimuli analogous to primary macrophages, making them a suitable model for studying the inflammatory response in endodontic scenarios.^32,33^

Essential to the pathogenesis of apical periodontitis, lipopolysaccharide is characterized as a pathogen-associated molecular pattern (PAMP) and a potent endotoxin found in the outer membrane of gram-negative bacteria that can cause a range of inflammatory responses by activating the TLR pathway, specifically TLR4, leading to the release of pro-inflammatory cytokines and chemokines.^34^ Therefore, evaluating the TLR pathway in an LPS-induced infection or inflammatory disease is critical since it can provide insights into the molecular mechanisms underlying the host’s response to bacterial infections and help identify the key signaling components involved in LPS recognition and the downstream inflammatory response, and can aid in developing targeted therapies aimed at modulating TLR signaling and mitigating the inflammatory response.

Activation of the NF-κB transcription factor is a subsequent event due to LPS binding to TLR4 and has been shown to modulate the expression of various pro-inflammatory cytokines, including upregulation of IL-1α, which is a potent pro-inflammatory cytokine that is involved in the pathogenesis of many inflammatory diseases.^35^ In addition, it induces the expression of IL-6, a pleiotropic cytokine involved in various physiological and pathological processes, including immune response, inflammation, and oncogenesis.^36^ IL-6 also activates NF-κB signaling, creating a positive feedback loop that amplifies the inflammatory response.^37^ Overall, the modulation of IL-1α, TNF-α, and IL-6 expression by NF-κB activation highlights the complex interplay between NF-κB signaling and the immune and inflammatory responses.^38^ In the literature, it is well established that sHDL inhibits the NF-κB pathway through multiple mechanisms. These mechanisms include interference with TLR signaling, lipid rafts modulation, cholesterol efflux promotion, anti-inflammatory and antioxidant properties, and direct interaction with NF-κB signaling components. These actions collectively contribute to the anti-inflammatory effects of sHDL and its potential therapeutic benefits in various inflammatory diseases.^18,39,40^ Through our luciferase assay, we appreciate the ability of sHDL to inhibit the NF-κB activation by LPS, as well as the diminished production of IL-6 by ELISA, which could also support and explain the less inflammatory profile found in vivo.

Furthermore, LPS can also directly influence the activity of osteoclasts, cells responsible for bone resorption. LPS can increase the expression of receptor activator of NF-κB ligand (RANKL) on osteoblasts, which promotes osteoclast differentiation and bone resorption.^41^ This can contribute to the bone loss often observed in apical periodontitis. An interesting finding in the present investigation was that the sHDL administration decreased osteoclast formation in vitro dose-dependently from 50 to 500 µg/mL, later confirmed by decreased lesion size through microtomography from our in vivo model. However, when we raised the concentration to 1 000 µg/mL, this reduction pattern reverted to a slight increase, although statistically significantly smaller than the group without sHDL. Such a phenomenon may be attributed to the cytotoxicity of sHDL at high concentrations. Our previous studies suggested that sHDLs had no negative effects on the viability of RAW264.7 cells with concentrations below 100 μg/mL.^42^ However, high sHDL concentrations, such as 1000 μg/mL, may lead to cytotoxicity due to excessive cholesterol efflux. Moreover, according to the literature, high cholesterol or HDL levels can activate inflammatory factors that increase bone turnover, leading to a decrease in bone mass, suggesting that the use of sHDL must depend on well-defined and precise concentrations so that it inactivates the LPS, hindering the RANKL binding to RANK and subsequent osteoclastogenesis.^43,44^

Our extensive gene expression evaluation through the TLR PCR array showed that several were upregulated when the sHDL was applied with the LPS. The most upregulated *Muc13* gene encodes a transmembrane mucin called Mucin 13, expressed in immune cells such as macrophages and dendritic cells. Although the precise function of Mucin 13 is not fully understood, evidence suggests that it regulates inflammation and promotes tissue repair by preserving the epithelial barrier’s integrity and preventing the entry of inflammatory agents such as LPS. This mechanism leads to decreased production of pro-inflammatory cytokines and chemokines, ultimately reducing inflammation.^45^

Another overexpressed gene was the interferon regulatory factor 3 (*Irf3*), a constitutively expressed transcription factor critical in the innate immune response to infections. *Irf3* activation leads to the production of type I interferons and promotes the activation of immune cells, including macrophages.^46,47^ Besides that, *Irf3* has been found to have anti-inflammatory abilities since *Irf3*-deficient mice showed exacerbated inflammation in various models of inflammatory diseases, indicating that its activation could limit inflammation.^48,49^ Additionally, *Irf3* has been implicated in regulating macrophage polarization, with *Irf3* activation favoring a shift toward an anti-inflammatory M2 phenotype. In contrast, loss of *Irf3* led to an increase in pro-inflammatory M1 macrophages. Therefore, *Irf3* not only plays a crucial role in immunity but also possesses anti-inflammatory abilities and macrophage polarization capabilities, making it a potential therapeutic target for inflammatory diseases.^49,50^

Also upregulated, the *Il12a* gene encodes a subunit of the cytokine Interleukin 12. While this gene is primarily known for up-regulating inflammatory responses, evidence suggests it also has anti-inflammatory properties, mainly inhibiting the proliferation of pro-inflammatory effector T helper cells. Furthermore, in vitro studies have shown that IL-12 can suppress the differentiation and function of mature osteoclasts induced by the RANKL. When looking at the overall pathogenesis of apical periodontitis in vivo, the endogenous effects of IL-12 do not appear significant.^51–54^

Contradictorily, the administration of sHDL increased the expression of *Irak1*, which stands for Interleukin-1 receptor-associated kinase 1, a serine/threonine protein kinase that plays a critical role in the immune response. This kinase is involved in the signaling pathways activated by TLRs and interleukin-1 receptors (IL-1Rs) that are present in immune cells and recognize PAMPs or damage-associated molecular patterns (DAMPs), triggering a cascade of signaling events that lead to the activation of transcription factors, such as the NF-κB pathway and the MAPK pathway, which culminate and the production of pro-inflammatory cytokines.^55^

Amongst the fourteen genes downregulated, the three most numerically and statistically significant genes were *Il1b*, *Il1a*, and *Ccl2*. IL-1β and IL-1α are strong pro-inflammatory cytokines regulating immune response and inflammation. Both IL-1β and IL-1α are produced by various cells, including immune cells such as macrophages and dendritic cells, in response to inflammatory stimuli such as bacterial or viral infections.^56^ The novelty of our approach was to use sHDL as a treatment for apical periodontitis. While our animal model exhibited no significant differences in the immunolabeling of IL-1β and IL-1α compared to the currently standardized treatment (NaOCl + Ca(OH)_2_), it effectively reduced the expression of both pro-inflammatory interleukins compared to untreated groups. Our PCR array analysis yielded similar results, with these two interleukins being the most downregulated, suggesting that the observed anti-inflammatory effect in histology is primarily attributable to the modulation of these two proteins compared to SHAM.

The *Ccl2* gene, which encodes the C-C motif chemokine ligand 2 protein (also known as monocyte chemoattractant protein-1 or MCP-1), is produced by a variety of cells including macrophages, endothelial cells, and smooth muscle cells, in response to pro-inflammatory stimuli such as cytokines and LPS. It has been found to promote chemotaxis, differentiation, activation of osteoclasts, and perpetuation of the inflammatory response. Moreover, a study has demonstrated that in the presence of RANKL, MCP-1 significantly increased the number of TRAP-positive multinuclear bone-resorbing osteoclasts in vitro,^57^ and a previous investigation using an in vivo model demonstrated the increased expression of MCP-1 in the apical lesion that can elicit pro-inflammatory cytokines, such as TNF-α and IL-1β, which can further perpetuate the inflammatory response and contribute to tissue destruction.^58^ This highlights the potential of sHDL since it targets the *Ccl2*, and its downstream effects may be a potential therapeutic approach for treating apical periodontitis.

Another explanation for how the sHDL promotes the anti-inflammatory effects can be linked to the upregulated gene expression of *Ppara* (Peroxisome Proliferator-Activated Receptor alpha) and *Fadd* (Fas-Associated protein with Death Domain) found in our PCR array. *Ppara* and *Fadd* play crucial roles in regulating inflammation, although through different mechanisms. *Ppara* is a nuclear receptor that modulates gene expression involved in lipid metabolism and inflammation by binding to c-Jun and to the p65 subunit of NF-kB. Activation of *Ppara* by fatty acids or synthetic agonists suppresses inflammatory responses by inhibiting the expression of pro-inflammatory genes and promoting the expression of anti-inflammatory genes.^59,60^ *Fadd*, known for its role in apoptosis signaling, may negatively regulate the TLR4-NFκB axis by interfering with MyD88 (myeloid differentiation primary response 88), a crucial adapter protein in TLR4 signaling. Upon TLR4 activation by ligands like LPS, MyD88 is recruited, initiating a cascade leading to NFκB activation and further pro-inflammatory cytokines like IL-1α and IL-1β production. FADD’s interaction with MyD88, likely facilitated by their death domains, could disrupt the formation of active signaling complexes necessary for NFκB activation, thus dampening the immune response. Additionally, FADD’s pro-apoptotic function might contribute to this negative regulation by promoting cell death, providing a potential feedback mechanism to prevent excessive or prolonged immune activation and inflammation-associated pathology.^61^ Overall, our findings demonstrated that sHDL could effectively control the inflammatory reaction and bone resorption associated with apical periodontitis by upregulating *Ppara* and *Fadd* and reducing the expression of proinflammatory cytokines, suggesting a partial inhibition of the TLR pathway.

Translating to the clinical approach, manually preparing calcium hydroxide before its application inside the root canal is a cost-effective option. However, in pursuit of enhanced ease of use, pre-mixed calcium hydroxide in syringes has entered the market as a more convenient but slightly pricier alternative. Also, calcium hydroxide injection directly into the periradicular area, a common occurrence in cases of immature apexes, perforations, or close proximity to neurovascular structures such as the inferior alveolar nerve and its mental distribution (typically associated with mandibular posterior teeth), poses severe risks to patients. The highly alkaline nature of calcium hydroxide can lead to inflammation and irreversible damage to surrounding tissues, including bone and soft tissues, resulting in complications such as paresthesia, dysesthesia, and necrosis. Furthermore, extruded calcium hydroxide lacks systemic absorption capability and may cause significant harm if it enters the bloodstream.^62^

In this way, our sHDL technology represents a novel and safer approach since it resembles an endogenously produced molecule of the body. Understandably, it comes with a higher price tag than existing materials. Nevertheless, as the application of this advanced technology becomes more widespread, manufacturing costs are expected to decrease, not adversely affecting the overall price of root canal treatments, ensuring accessibility and affordability for patients. Our findings shed a green light on the potential use of pure HDL-like carrier as a therapeutic agent for apical periodontitis and pave the way to improve it by loading the nanoparticle core with drugs or other therapeutic agents that target specific tissues or cells, potentially enhancing the efficacy of certain medications while minimizing side effects. Since this is the first study evaluating this lipoprotein for endodontic treatment, we can point out some limitations, such as using a small animal model and the absence of sHDL residence and stability time assessment when locally administered to teeth. Thus, further studies that evaluate these parameters coupled to a larger animal model, such as canine or porcine, are crucial to bringing us closer to replicating a clinical scenario in human teeth and refining carrier systems to enhance the delivery of sHDL.

## Methods

### Preparation and characterization of synthetic high-density lipoprotein

The 22A-DMPC sHDL was composed of an ApoA-1 mimetic peptide 22A (PVLDLFRELLNELLEALKQKLK) synthesized by Genscript (Piscataway, NJ) and 1,2-dimyristoyl-sn-glycero-3-phosphocholine (DMPC) from NOF America Corporation (White Plains, Nova York). The sHDL was prepared by a co-lyophilization procedure where peptide and phospholipids were dissolved in glacial acetic acid, mixed at a 1:2 w/w ratio of peptide/lipid, and lyophilized overnight. The powder was hydrated with phosphate-buffered saline (PBS; pH 7.4) to make 10 mg/mL sHDL and cycled between 55 °C (10 min) and room temperature (10 min) to facilitate the sHDL formation.^63,64^ The particle size of sHDL was determined by dynamic light scattering (DLS) on Malvern Zetasizer Nano ZSP (Westborough, MA). The particle size distribution of sHDL was further examined by gel permeation chromatography (GPC) on a Tosoh TSK gel G3000SWxl column with a PBS flow rate of 0.75 mL/min and UV detection at 220 nm. The morphology of sHDL particles was imaged by transmission electron microscopy (TEM) imaging. sHDL was loaded on a carbon film-coated 400 mesh copper grid from Electron Microscopy Sciences (Hatfield, PA), negatively stained with 1% (w/v) uranyl formate, and imaged with 100 kV Morgagni TEM after drying. To evaluate the particle stability of sHDL nanoparticles, the particle size of sHDL nanoparticles after 30 min or 12 h incubation at 37 °C was determined by DLS.

### NF-κB reporter assay

The effect of sHDL nanoparticles per se or in the presence of LPS on nuclear factor Kappa B (NF-κB) signaling transduction pathway was assessed by a reporter assay using the macrophage-like cell NF-κB Luciferase Stable RAW264.7 cells (T3015, Applied Biological Materials Inc., Canada) where the luciferase expression depends on NF-κB promoter activation. The cells were seeded at 0.75 ×10^5^ cells per well in 96-well plates with Dulbecco’s Modified Eagle Medium (DMEM) (#11965, Gibco, Rockville, MD, USA) supplemented with 10% Fetal Bovine Serum (FBS) (#10438026, Gibco). Later, the cells were cultured with sHDL at increasing concentrations (25, 50, 75, 100, 500, and 1 000 µg/mL) without or in the presence of 0.05 µg/mL of Escherichia coli–LPS serotype 0111:B4 (Sigma-Aldrich, USA). The level of NF-κB activation was determined at 6 hours post-stimulation using a luciferase assay system (Promega, USA) performing a 2-second measurement delay followed by a 10-second measurement read for luciferase activity with the SpectraMax iD3 plate reader (Molecular Devices, USA).

### RANKL-induced osteoclastogenesis

RAW264.7 cells were seeded in 48-well plates at 3×10^3^ cells/well density in DMEM containing 10% FBS. After adhered, the cells were cultivated with 50 ng/mL RANKL (#462-TR/R&D system, USA) alone or in the presence of sHDL (50, 100, 500, and 1 000 µg/mL). The media was replaced every two days. Five days later, the cells were stained for tartrate-resistant acid phosphatase (TRAP) using a leukocyte acid phosphatase kit (Sigma-Aldrich, USA). TRAP+ cells with more than three nuclei were considered osteoclasts and counted, and the results were expressed as mean number and standard deviation per group.^65^

### Enzyme-linked immunosorbent assay

RAW264.7 cells were seeded at 0.75 ×10^5^ cells per well in 96-well plates harvested in DMEM containing 10% FBS and were stimulated with 0.05 µg/mL LPS without or in the presence of sHDL (50, 100, 500, 1 000 µg/mL) for 24 h. Medium alone served as a control. The effect of sHDL nanoparticles per se or in the presence of LPS on the expression of IL-1α, TNF-α, and IL-6 in cell culture supernatants was determined by ELISA with commercially available kits (#433404, #431304, and #430904, Biolegend, USA) following the manufacturers’ instructions.

### PCR array - toll-like receptor signaling pathway

RAW264.7 cells were seeded at 0.3 ×10^6^ per well in 6-well plates with DMEM supplemented with 10% FBS and were stimulated with 0.1 µg/mL LPS without or in the presence of 500 µg/mL of sHDL for 24 h. The TRIzol™ Plus RNA Purification Kit (Invitrogen Life Technologies, Carlsbad, CA, USA), a combination of TRIzol™ Reagent and PureLink™ RNA Mini Kit, was used to isolate and purify the total RNA from the cultured cells. The concentration and purity of the isolated RNA were examined using a spectrophotometer (SpectraMax iD3). The complementary DNA (cDNA) was synthesized from 0.5 μg of total RNA using RT2 First Strand Kit (Qiagen, Germantown, MD, USA) according to the manufacturer’s instructions. The reactions were performed in the MiniAmp™ Plus Thermal Cycler (Applied Biosystems, Waltham, Massachusetts, USA) and stored at −20°C until use. Next, the cDNA was mixed with the RT^2^ SYBR® Green ROX qPCR Mastermix. The Toll-Like Receptor Signaling Pathway PCR array was performed with cDNAs as templates using RT2 Profiler™ PCR Array Mouse Toll-Like Receptor Signaling Pathway (Cat. no. 330231 PAMM-018ZA, Qiagen) and RT2 SYBR Green qPCR Mastermix (Qiagen). The TLR PCR Array comprises 84 pathway-focused genes, 5 housekeeping genes, one well containing a genomic DNA control, 3 wells containing reverse-transcription controls, and 3 wells containing positive PCR controls. To conduct a quantitative RT-PCR, the 7500 Real-Time PCR System (Applied Biosystems) was used to amplify the cDNA. The data analysis was conducted at QIAGEN’S GeneGlobe Data Analysis Center using a software-based tool using the ΔΔCt method to calculate the relative gene expression and fold change. Quality control was confirmed with the positive PCR controls and reverse transcription controls of the PCR array. Only the results that passed quality checks in PCR array reproducibility and the lack of genomic DNA contamination were included. The fold changes were expressed as Log_2_ to mitigate and facilitate the heatmap interpretation, and a volcano plot was created by plotting the Log_2_ fold changes in gene expression between LPS + sHDL vs LPS against t-test p-values. Changes in gene expression were considered significant if the detection *P*-value was <0.05 and the fold change value was >2.0.

### Animals

A group of thirty adult male Fischer 344 rats (Envigo RMS, USA), with similar age and weight (250–300 g), were housed under controlled conditions of temperature (22 °C ± 1 °C) and humidity (70%). The rats were kept in a 12-h light-dark cycle and provided unlimited water and food access. All experimental protocols were approved by the Institutional Animal Care and Use Committee (PRO00010911) and followed the appropriate guidelines. The apical periodontitis induction was performed under general anesthesia with 50 mg/kg of ketamine (Hospira, USA) and 5 mg/kg xylazine (Akorn, USA) intraperitoneally. Briefly, the dental pulp on the central portion of the occlusal surface in the lower first molars was exposed through endodontic access using ¼ dental round burr (Kavo Kerr Group, USA). A #25 endodontic file (Dentsply Maillefer, Switzerland) was initially used to disorganize the pulp tissue and perform the endodontic instrumentation at a later root canal disinfection stage. The cavities were left open, allowing contamination of root canals with the oral commensal microorganisms for three weeks to induce the periapical lesion. The animals were divided into five groups: (1) Control group - no endodontic treatment performed; (2) NaOCl – root canal instrumentation and 5.25% sodium hypochlorite irrigation; (3) NaOCl + Calcium Hydroxide − root canal instrumentation, 5.25% sodium hypochlorite irrigation and UltraCal XS calcium hydroxide paste application (Ultradent, USA); (4) NaOCl + sHDL − root canal instrumentation, 5.25% sodium hypochlorite irrigation, and 20 µL of 500 µg/mL sHDL; and (5) PBS + sHDL − root canal instrumentation, PBS irrigation, and 20 µL of 500 µg/mL sHDL. Right before calcium hydroxide or sHDL solution placement, the canals were dried with sterile paper points. The teeth were sealed for all groups with a combination of glass ionomer (Fuji II, GC, Tokyo, Japan) and temporary sealing material Coltosol (Coltene, Cuyahoga Falls, OH, USA). Four weeks post-treatment, the animals were euthanized by the inhalation of carbon dioxide. The right and left sides of the jaws were removed and stored in a 10% buffered formaldehyde solution.

### Micro–computed tomography scanning

Fixed hemimandibles were scanned using a cone beam-type tomograph (SCANCO Medical μCT 100, SCANCO Medical AG, Switzerland).^65,66^ To quantify the periradicular bone resorption area, a region of interest (ROI) was drawn around the root and apical foramen, starting from the first transaxial cut showing bone resorption, continuing through the apex of the root, ending at the final slice where the normal bone resumes.

### Histological and immunohistochemical analyses

After the µCT scanning, the hemimandibles were decalcified using 10% ethylenediaminetetraacetic acid (EDTA) for 60 days. Decalcified samples were dehydrated in graded alcohols, embedded in paraffin, and sectioned at 6 μm thickness. Haematoxylin and eosin (H&E) staining was used to analyze the inflammatory profile and condition of the periapical region. The inflammatory cells were counted using the ImageJ software (U. S. National Institutes of Health, Bethesda, Maryland, USA) at 10X magnification. Briefly, three distinct images (around the tooth apex) per sample were converted from the original image to grayscale by adjusting its color channels to an 8-bit scale. Next, the images were thresholded using an optimal threshold value to differentiate inflammatory cells from the background. Once the threshold was applied, the images were analyzed for particle count, and specific size and circularity criteria were set by adjusting the respective values in the Analyze Particles dialog box.^67^

For the immunohistochemical analysis, the sections were dewaxed at 60 °C for 15 min, rehydrated using a gradient of ethanol, then incubated in 3% hydrogen peroxide at room temperature (20 min) to reduce the activity of endogenous peroxidase, followed by an additional 20-min incubation in Background Sniper (Biocare Medical, USA) to block unspecific binding. Based on the PCR array results, which showed the highest positive fold regulation for IL-1α and IL-1β, we decided to immunolabel these interleukins in vivo, using an anti-IL-1 alpha antibody (1:40, ab7632, Abcam) and anti-IL-1 beta antibody (1:500, ab283818, Abcam) overnight. The goat anti-rabbit IgG H&L (HRP) (1:200, ab97051, Abcam) was used as the secondary antibody. After washing, slides were incubated with a DAB (3,3′-diaminobenzidine) substrate kit (ab64238, Abcam) and immediately washed under tap water after color development. Slides were then counter-stained with hematoxylin QS (H-3404, Vector Laboratories, Newark, California, USA) and mounted with VectaMount® Express Mounting Medium (H-5700, Vector Laboratories). To evaluate the specificity of the immunostaining process, sections were processed under the same conditions as the experimental samples, except for omitting the primary antibody from the protocol. The images were captured using an Aperio ScanScope slide scanner (Leica Biosystems, Wetzlar, Germany), and ImageJ software was used to calculate the percentage of DAB-positive area per tissue section.

### Statistical analysis

The data were analyzed using Prism 9 software (GraphPad). After the normality test, the parametric data were subjected to a one-way analysis of variance with Tukey’s multiple comparisons test. The level of significance was 5%.

## Data Availability

All data associated with this study are presented in the paper.
